# Assessing health status in COPD. A head-to-head comparison between the COPD assessment test (CAT) and the clinical COPD questionnaire (CCQ)

**DOI:** 10.1186/1471-2466-12-20

**Published:** 2012-05-20

**Authors:** Ioanna G Tsiligianni, Thys van der Molen, Despoina Moraitaki, Ilaine Lopez, Janwillem WH Kocks, Konstantinos Karagiannis, Nikolaos Siafakas, Nikolaos Tzanakis

**Affiliations:** 1 Department of Thoracic Medicine, Medical School, University of Crete, Heraklion, Crete, P.O 71003, Greece; 2 Department of General Practice, University Medical Centre Groningen, Antonius Deusinglaan 1, P.O 9700 AD, Groningen, The Netherlands; 3 GRIAC research institute, University Medical Center Groningen, University of Groningen, Antonius Deusinglaan 1, P.O 9700 AD, Groningen, The Netherlands

**Keywords:** Health status, COPD Assessment Test (CAT), Clinical COPD Questionnaire (CCQ)

## Abstract

**Background:**

Health status provides valuable information, complementary to spirometry and improvement of health status has become an important treatment goal in COPD management. We compared the usefulness and validity of the COPD Assessment Test (CAT) and the Clinical COPD Questionnaire (CCQ), two simple questionnaires, in comparison with the St. George Respiratory Questionnaire (SGRQ).

**Methods:**

We administered the CAT, CCQ and SGRQ in patients with COPD stage I-IV during three visits. Spirometry, 6 MWT, MRC scale, BODE index, and patients perspectives on questionnaires were recorded in all visits. Standard Error of Measurement (SEM) was used to calculate the Minimal Clinical Important Difference (MCID) of all questionnaires.

**Results:**

We enrolled 90 COPD patients. Cronbach's alpha for both CAT and CCQ was high (0.86 and 0.89, respectively). Patients with severe COPD reported worse health status compared to milder subgroups. CAT and CCQ correlated significantly (rho =0.64, p < 0.01) and both with the SGRQ (rho = 0.65; CAT and rho = 0.77; CCQ, p < 0.01). Both questionnaires exhibited a weak correlation with lung function (rho = −0.35;CAT and rho = −0.41; CCQ, p < 0.01). Their reproducibility was high; CAT: ICC = 0.94 (CI 0.92-0.96), total CCQ ICC = 0.95 (0.92-0.96) and SGRQ = 0.97 (CI 0.95-0.98). The MCID calculated using the SEM method showed results similar to previous studies of 3.76 for the CAT, 0.41 for the CCQ and 4.84 for SGRQ. Patients suggested both CAT and CCQ as easier tools than SGRQ in terms of complexity and time considerations. More than half of patients preferred CCQ instead of CAT.

**Conclusions:**

The CAT and CCQ have similar psychometric properties with a slight advantage for CCQ based mainly on patients’ preference and are both valid and reliable questionnaires to assess health status in COPD patients.

## Background

Chronic Obstructive Pulmonary Disease (COPD) is a prevalent disease in the general population and it has been estimated that it will be the fourth leading cause of death by the end of 2030 [1]. Apart of its high mortality, one main concern for physicians is that COPD strongly impairs health status and quality of life. Quality of life is an important goal in COPD management that has been highlighted as a future research need from the recent International Primary Care Respiratory Group (IPCRG) research needs statement [2].

Patients with COPD often develop symptoms as dyspnoea, cough, phlegm, chest tightness, exercise intolerance, sleep and mental disorders as well as restriction of social activities. In every day practice COPD treatment and management guidance is currently largely based on the spirometric assessment. Recently, GOLD guidelines proposed health status, dyspnea measurement and number of exacerbations as key elements in addition to spirometry in order to manage and treat COPD [3]. This is mainly based on the fact that spirometry is only weakly associated with various health status questionnaires and does not give a real image of the COPD patients wellbeing [4]. Numerous quality of life and/or health status questionnaire tools have been developed in an attempt to find an easy and reliable tool to use in every day clinical practice [5-10]. Even though most COPD-specific health status questionnaires show similar basic content, there is heterogeneity in the amount and quality of the items addressed [11].

The IPCRG has recently published a ‘users guide to COPD wellness tools’ in order to provide physicians with the available questionnaires and rank them in terms of validity, reliability, responsiveness, usefulness in a primary care population, practicality and tested in practice [12]. From this wellness guide, both CAT and CCQ are the preferred questionnaires compared to the SGRQ that has been traditionally used as the gold standard for the assessment of health status in COPD. Although SGRQ reflects very well the COPD health status it is rather complicated, time consuming and requires complicated spreadsheets to calculate the scores [5]. On the other hand the CAT and CCQ are practical, easy to use, and can be completed in 2 minutes at most. Both have been designed for use in primary care population, they are self-completed, available in many translations and free of charge either for clinicians or patients [9,10,12]. CAT is the newest one developed in 2009 [10], while CCQ has been widely used since its development in 2003 [9]. The ‘IPCRG COPD users guide to wellness tools’ has ranked CCQ as best and CAT as second best for use in daily practice [12].

Both CAT and CCQ have been suggested as tools to measure health status in daily clinical practice in COPD patients but there has not been a study comparing these two questionnaires in every-day practice. The current study aimed to make a head-to-head comparison between the two questionnaires (CAT and CCQ) in order to help physicians to choose the tool that meets their needs taking in consideration psychometric properties and patient’s preference.

## Methods

### Subjects

Subjects participating were primary and secondary care patients diagnosed with COPD in Crete, Greece. We included patients 45 years of age and older with a smoking history of at least 10 years. Exclusion criteria were patients with concomitant asthma, unstable cardiovascular disease or any other respiratory disease other than COPD. GOLD guidelines were used to classify disease severity [3]. We approached 101 patients. Eleven patients did not complete the study (one died after the 2nd visit, one did not meet the inclusion criteria and 9 patients were lost to follow-up). 90 patients completed all three visits. The study was approved by the local medical ethics committee of the University Hospital of Crete, Greece and the patients gave written informed consent. The study took place from July 2010 to June 2011.

### Data collection

In order to assess the test-retest reliability of the CAT and CCQ questionnaires, CAT, CCQ and SGRQ were re-administered during two subsequent visits, at baseline and after 2 and 6 weeks from baseline.

Demographic information and medical history were recorded. Baseline spirometry was performed during each visit using a Microlab 2000 spirometer, Jaeger Germany, including post-bronchodilator lung function 20 minutes after inhalation of 400 mcg salbutamol. GOLD criteria for COPD were followed. COPD diagnosis was based on chest physician examination including spirometry test after bronchodialtion with FEV1/FVC ratio lower than 0.70. Pulmonary function predicted values were obtained from the standardized lung function testing of the European Community for Steel and Coal Luxembourg 1993 (ECSC) [13]. Body mass index (BMI), the 6-minute walking test (6MWT), the Medical Research Council dyspnoea scale (MRC) [14] and pulse oxymetry before and after the 6MWT were assessed during each visit. Scores on the BODE-index {body mass index, airflow limitation (forced expiratory volume in one second), dyspnoea and 6-min walk distance} were also divided into four quartiles [15]. Quartile 1 contains score 0–2, quartile 2 contains score 3–4, quartile 3 contains score 5–6 and quartile 4 contains score 7–10 [15].

### Health status questionnaires

The St. George Respiratory Questionnaire (SGRQ) [5], the COPD Assessment Test (CAT) [10] and the Clinical COPD Questionnaire (CCQ) [9] were administered to all subjects during each visit in a different order for each visit in each patient. All patients administered the Global Rating of Change scale in visits 2 and 3 (GRC) [16].

The SGRQ is a 50-item questionnaire. Three component scores are calculated: symptoms, activity, impacts (on daily life), and a total score [5]. The CAT has 8 items and raise questions like symptoms, energy, sleep and activity [10]. The CCQ contains 10 items, divided into 3 domains (symptoms, functional and mental state) [9]. The GRC used was a 7-point Likert scale ranging from much better to much worse.

### Patients view on questionnaires

A qualitative approach was used, in which patients were asked by simple open-ended questions to express their opinion on which questionnaire was easier to complete in terms of complexity and time needed to fill out, as well as which reflected better their personal well-being.

### Statistical analysis

The statistical analysis was performed using SPSS for Windows version 18 (SPSS Inc. IL, USA). Data are expressed as median (interquartile range) unless otherwise stated. We used the Kruskal-Wallis test for normally distributed continuous data, and the Chi-square test for not normally distributed continuous data and categorical data. Normality of the data was evaluated using the Kolmogorov-Smirnov and Sapiro-Wilk test. CCQ and CAT internal consistency was evaluated by calculating Cronbach’s alpha coefficient.

Discriminant validity of the CAT and CCQ was determined with the non-parametric Kruskal-Wallis test in COPD GOLD stages I-IV, we subsequently used the Mann–Whitney *U* test to compare specific groups. Test-retest reliability was assessed by calculating the Intra-class Correlation Coefficient (ICC). Convergent and divergent validity were examined using Spearman's rank correlations. Responsiveness of both the CAT and CCQ was determined using the Wilcoxon *U* test. A value of p < 0.05 was considered as statistically significant. The Minimal Clinically Important Difference (MCID) for the CAT and CCQ, the smallest calculated change in score that is perceived as relevant was assessed by using the GRC and the Standard Error of Measurement (SEM).

In order to calculate the SEM of the CAT CCQ and SGRQ questionnaires we used [17] SEM = σx √1-rxx σx = standard deviation of the questionnaire at baseline rxx = the reliability/Intraclass Correlation Coefficient (ICC) of the questionnaire.

Bland and Altman graphs were made to assess the agreement between questionnaires. This technique compares the scores of two measurements across the entire scaling range. Because the SGRQ, CAT and CCQ all have different scaling ranges, CAT and CCQ scores were transformed to a maximum score of 100 similar to the SGRQ range. CAT scores were multiplied by 2.5 (100/40) and CCQ scores by 16.67 (100/6). The adjusted scores were named adjCAT and adjCCQ.

## Results

### Patient demographics

A total of 90 patients completed the study. The median age of the patients was 67 years (58–75 years), 90% were male. The characteristics of our study population are displayed in Table 1. We found no differences among GOLD severity stages in terms of age, gender, BMI and pack-years smoking (Table 1).

**Table 1:** Characteristics of the study population at baseline (n = 90)

**COPD GOLD stage**					
	Stage I(mild)	Stage II (moderate)	Stage III (severe)	Stage IV (very severe)	P-value
N	15	42	27	6	
Males (%)	86.7	86	96.3	100	0.41
Age (years)	68 (58–74)	67 (62–73)	68 (61–73)	63 (53–75)	0.78
Pack years	60 (42–75)	55 (34–81)	70 (40–92)	68 (50–151)	0.52
BMI (kg/m²)	27 (26–32)	29 (25–34)	26 (24–30)	25 (21–29)	0.12
Current smoking (%)	53.3	52.4	51.9	33.3	0.84
FEV_1_ (% predicted)	83 (81–86)	62 (56–70)	37 (33–44)	22 (20–27)	p < 0.01
6MWD (m)	450 (360–480)	420 (315–545)	360 (300–420)	375 (255–458)	0.07
MRC dyspnea grade	1 (1–2)	1 (1–2)	2 (1–3)	3 (2–4)	P < 0.01
BODE index quartile					
(n(%))					
1	15 (100.0)	40 (95.2)	8 (29.6)	1 (16.7)	
2		1 (2.4)	14 (51.9)	1 (16.7)	
3		1 (2.4)	4 (14.8)	3 (50.0)	
4			1 (3.7)	1 (16.7)	

Data represent median (interquartile range). BMI = body mass index. Pack years: amount of cigarette packs smoked per day multiplied by the amount of years smoked. 6MWD = 6 Minute Walking Distance. MRC = Medical Research Council dyspnoea scale. BODE: body mass index, airflow limitation (forced expiratory volume in one second), dyspnoea and 6-min walk distance. FEV1 = Post-bronchodilator Forced Expiratory Volume in 1 s 20 min after inhaled bronchodilator. GOLD stages = COPD classification by post-bronchodilator spirometry according to GOLD guidelines.

### Health status questionnaires-GOLD stage

**Figure 1:**
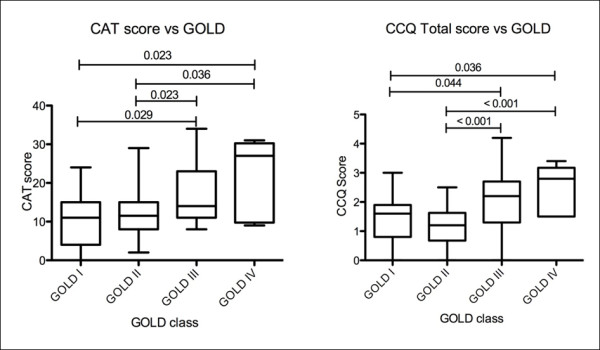
**Numbers above lines represent p values.** COPD Assessment Test (CAT) and Clinical COPD Questionnaire (CCQ) scores for GOLD COPD stages.

Health status by COPD GOLD stage according to CAT, CCQ and SGRQ is depicted in Figure1.

### Relationship between questionnaires

**Figure 2:**
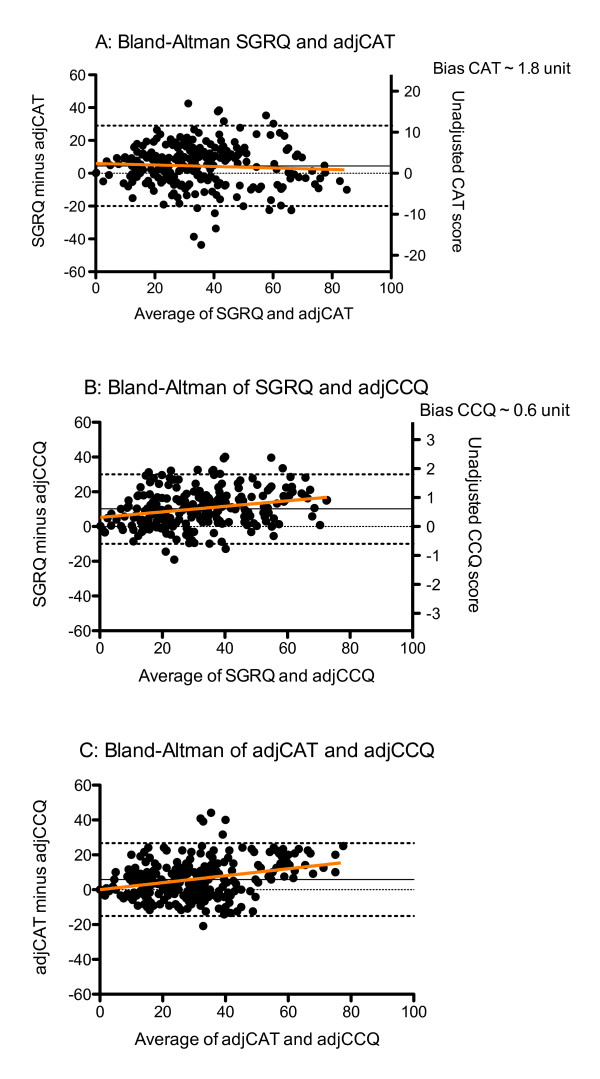
**Bland and Altman plots for SGRQ, CAT, CCQ.** SGRQ = St George’s Respiratory Questionnaire, CAT = COPD Assessment Test, CCQ = Clinical COPD Questionnaire. CAT scores are multiplied by 2.5(adjCAT), CCQ scores by 17.67(adjCCQ). Right Y ax shows the original scale. Orange line is the regression line. Dashed lines represent the 95% confidence interval. Straight line represents the bias.

**Figure 3:**
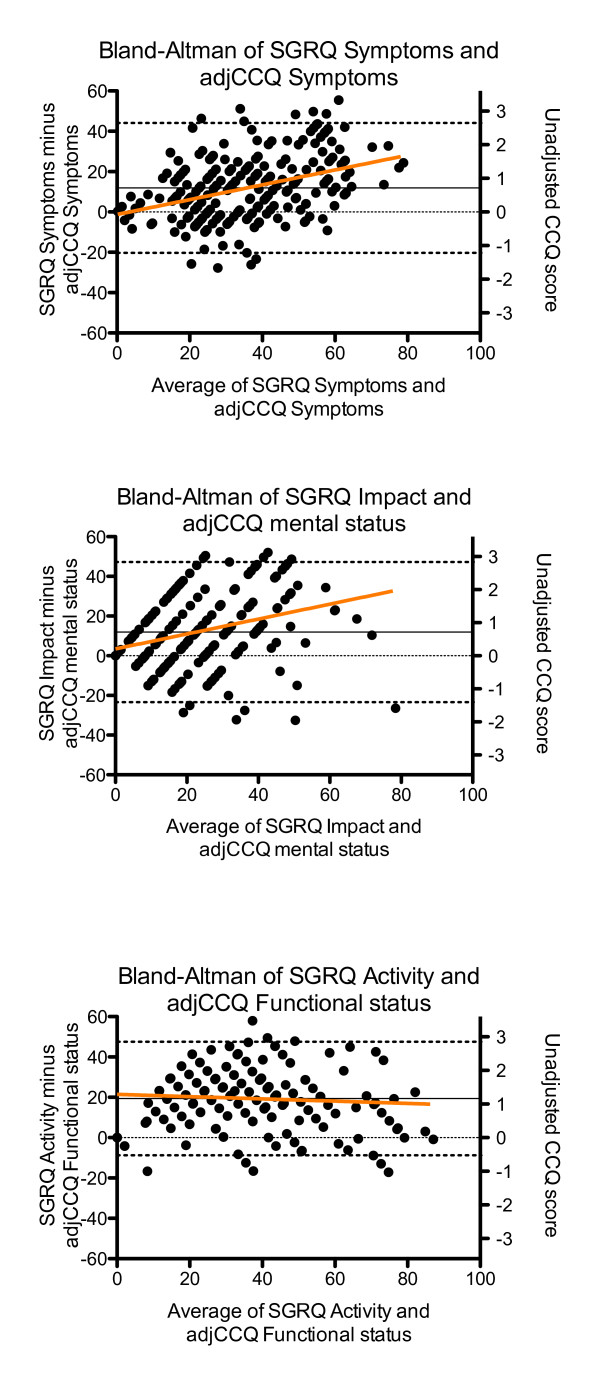
**Bland and Altman plots for the relation between subdomains of the SGRQ and CCQ.** SGRQ = St George’s Respiratory Questionnaire, CCQ = Clinical COPD Questionnaire. CCQ scores by 17.67(adjCCQ). Right Y ax shows the original CCQ scale. Orange line is the regression line. Dashed lines represent the 95% confidence interval. Straight line represents the bias.

The Bland and Altman plots reveal a stable relationship between the SGRQ and the CAT, with a mean bias of 1.8 CAT units. The relationship between SGRQ and CCQ show that the adjusted CCQ scores are lower across the scaling range and increasing with increasing health status impairment, with a mean bias of 0.6 CCQ units. The CAT CCQ plot shows that with decreasing health status, CAT scores are higher than CCQ scores (Figure2). Figure 3 shows the Bland and Altman plots for the SGRQ domains symptoms, activity and impact, compared with the CCQ domains sumptoms, functional status and mental status respectively. The CAT does not have domain scores.

### Construct validity

#### Internal consistency

Cronbach’s alpha was 0.86 for the CAT score and 0.89 for the CCQ total score. Internal consistencies for the symptom, mental state and functional state domain of the CCQ were 0.71, 0.71 and 0.90 respectively.

### GOLD stages

We compared CAT and CCQ between all COPD GOLD stages, and both questionnaires showed significant different scores between GOLD stages (Figure1). Patients with severe COPD (stage III) showed significantly higher CAT and CCQ total scores compared to the patients with mild disease (stage I). More details are depicted in Figure1.

### BODE severity-index

**Figure 4:**
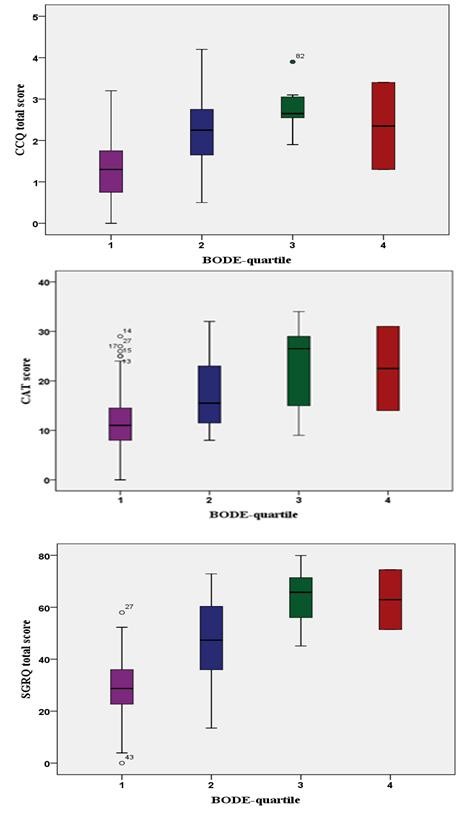
**Box plot showing the distribution of the CAT, CCQ and SGRQ total scores, grouped by BODE-quartiles.** SGRQ = St George’s Respiratory Questionnaire, CAT = COPD Assessment Test, CCQ = Clinical COPD Questionnaire. Horizontal bars represent median. Statistical significance between all three QoL scales: CCQ (p < 0.0001), CAT (p < 0.001) and SGRQ (p < 0.001).

Patients in BODE-quartiles 3 had worse CAT and CCQ scores than patients in the other quartiles. CAT, CCQ and SGRQ scores differed significantly among the BODE-quartiles (Figure4).

### Convergent validity

CAT, CCQ and SGRQ were strongly interrelated; correlations are depicted in Table 2. Further details are given on MRC and BODE index correlations with the questionnaires.

**Table 2:** Spearman rank correlations between health status questionnaires (CAT, CCQ, SGRQ), lung function, GOLD stage, MRC dyspnea scale, 6MWT and BODE-index at baseline

	**CAT**	**CCQ Total**	**CCQ Symptom**	**CCQ Mental**	**CCQ Functional**	**SGRQ Total**	**SGRQ Symptom**	**SGRQ Activity**	**SGRQ Impact**	**GOLD stage**	**FEV1% pred**
CAT	1	0.644**	0.540**	0.438**	0.616**	0.646**	0.404**	0.629**	0.662**	0.337**	−0.353**
CCQ											
Total	0.644**	1	0.867**	0.700**	0.932**	0.769**	0.502**	0.711**	0.703**	0.361**	−0.410**
Symptom	0.540**	0.867**	1	0.542**	0.690**	0.629**	0.584**	0.516**	0.590**	0.275**	−0.286**
Mental	0.438**	0.700**	0.542**	1	0.557**	0.529**	0.463**	0.448**	0.453**	0.222*	−0.298**
Functional	0.616**	0.932**	0.690**	0.557**	1	0.733**	0.366**	0.753**	0.660**	0.376**	−0.421**
SGRQ											
Total	0.646**	0.769**	0.629**	0.529**	0.733**	1	0.602**	0.830**	0.964**	0.474**	−0.487**
Symptom	0.404**	0.502**	0.584**	0.463**	0.366**	0.602**	1	0.322**	0.533**	0.357**	−0.357**
Activity	0.629**	0.711**	0.516**	0.448**	0.753**	0.830**	0.322**	1	0.740**	0.388**	−0.403**
Impact	0.662**	0.703**	0.590**	0.453**	0.660**	0.964**	0.533**	0.740**	1	0.450**	−0.443**
MRC	0.605**	0.739**	0.518**	0.435**	0.784**	0.690**	0.360**	0.736**	0.627**	0.372**	−0.437**
dyspnea											
Scale											
6MWT	−0.205(ns)	−0.239*	−0.170 (ns)	−0.071(ns)	−0.302**	−0.281**	−0.054 (ns)	−0.365**	−0.264*	−0.255*	0.216**
BODE-index	0.483**	0.556**	0.433**	0.347**	0.545**	0.609**	0.413**	0.524**	0.577**	0.732**	−0.705**

*Correlations are significant at the 0.05 level ** Correlations are significant at the 0.01 level. ns = not significant. Lung function is expressed as FEV1%predicted. CAT = COPD assessment Test. CCQ = Clinical COPD Questionnnaire. SGRQ = St. George Respiratory Questionnaire. 6MWT = 6-minute walking test. MRC = Medical Research Council dyspnoea scale, BODE = body mass index, airflow limitation (forced expiratory volume in one second), dyspnoea and 6-min walk distance.

### Divergent validity

Results on questionnaires total scores and domains and their correlation with FEV1%pred are depicted in Table 2.

### Longitudinal validity

#### Test-retest reliability

The Intraclass Correlation Coefficient for subsequent measures of all questionnaires were high; for CAT ICC = 0.94 (CI 0.92-0.96),for total CCQ ICC = 0.95 (CI 0.92-0.96) and for SGRQ = 0.97 (CI 0.95-0.98).

### Responsiveness

#### Overall change in scores

Patients in this study had stable disease without exacerbations and with no FEV1 changes. We found no significant changes in health status as assessed with the CAT, CCQ and SGRQ among all 3 visits.

### Minimal clinically important difference

#### Anchor-based approach/global rating of change

Since the number of patients reporting a change with the Global rating of change scale was low (2 and 7 patients at visit 2 or 3, respectively) it was not appropriate to determine the MCID using this approach.

#### Distribution-based approach-standard error of measurement

We used 1.96*SEM to calculate the MCID [17], this gives a MCID of 3.76 (SEM:1.92) for the CAT, 0.41 (SEM 0.21) for the CCQ and 4.84 for the SGRQ (SEM:2.47).

### Patients views on questionnaires (qualitative approach)

All patients (100%) perceived the CAT and CCQ as more easy tools compared to SGRQ in terms of complexity and time to complete. The SGRQ was considered rather complicated and time consuming. On the question ‘which tool, CAT or CCQ, would you select for assessing your health status?’ 61.1% (55 patients) expressed the opinion that the CCQ reflected their status better than CAT as it had more details on breathing problems which was more important for them than sleep or energy. Ten patients also expressed their opinion that the CCQ has a more easy to understand response option system as compared to the CAT. The other patients did not make any comments.

## Discussion

This study showed that both CAT and CCQ exhibit excellent reliability, good discriminate validity and high reproducibility. Both questionnaires can be used as easy and reliable tools to assess health status in COPD patients in studies as well as in daily clinical practice. Patients however preferred the CCQ since it reflected their health status better than the CAT.

The most widely used questionnaire for measuring health status in COPD in a research setting is the SGRQ. The main disadvantage for clinical practice is it’s extent as it comprises 50 questions and scores can only be calculated using a computer-based scoring system. This is in accordance with the patient’s views that perceived the SGRQ as rather complicated and time consuming. Daudey et al. based on empirical data proposed that SGRQ is not able to provide a detailed measurement of health status giving information mainly only in subjective symptoms and impairment [18].

The CAT and CCQ were designed to measure health status in COPD patients in clinical practice and are much shorter and easy to understand. Both can be instantly calculated. Indeed patients in our study found that both are pretty easy and reflect well their status. The response option of CCQ was more clear for patients than the CAT rank system and patients thought that CCQ better reflects their health status. An advantage of CCQ is that it has been validated to be used in individual patients [19]. In the above study patients were asked to fill in the CCQ and their results were compared to the opinion of clinicians who had seen the transcripts of an in depth interview with the same patients. The CCQ outcome of patients and clinicians was similar, supporting the individual validity.

The agreement between the questionnaire scores as reflected using Bland and Altman plot is high. The CCQ scores are generally lower at the higher end of the scales. For the comparison of the questionnaires, the scores had to be adjusted to a score of 100. The CCQ scores were multiplied by 16.67 for that purpose. A small difference in score magnifies using this calculation method. For the interpretation of the results, calculating the difference to the original scale reveals that the differences can hardly be considered clinical relevant. For example, a CAT score of 13 (median CAT score in this study) shows a difference in adjCCQ of 2.82. This represents a difference in original CCQ score of 0.17 or CAT score of 1.13. These findings are in line with previous SGRQ/CATcomparisons [20].

Our study showed that CAT and CCQ are both reliable questionnaires in terms of internal consistency for measuring health status in COPD patients. Their high Cronbach’s alpha (α = 0.86 for CAT and α = 0.89 for CCQ) indicate that there is homogeneity among the individual items in the questionnaires.

In terms of discriminant validity both CAT and CCQ showed a tendency to reflect the differences in COPD severity. Patients with more severe stages of COPD reported worse health status, measured with both CAT and CCQ similarly to other studies [21,22]. This is true for both severity scales GOLD and BODE used in this study. In order to examine if there is a type-1 statistical error, because of the small numbers in stages I & IV, we compared CAT and CCQ scores in COPD patients GOLD stage I & II subgroup with those of stages III & IV (data not shown). Although the statistical significance difference in these comparisons remains larger studies are needed to confirm these observations. Even though FEV1 was associated with health status in this study, correlations were only weak to modest. This was expected as the pulmonary function itself measured by FEV1, on which the GOLD classification of COPD stage is based, is not a good predictor of health status [4]. These results are in keeping with findings in previous studies (CCQ; rho = −0.49 and rho = −0.57, CAT; rho = −0.23) [9,21,22].

Our study is the first study that assessed the variation of all three questionnaires in BODE quartiles. The BODE-index is a grading system developed to predict mortality in COPD [15]. We found a great variation of health status in each BODE-quartile and surprisingly patients in the 3rd BODE-quartile reported worse health status as assessed with all questionnaires CAT, CCQ, SGRQ than patients in the 4th quartile. An explanation is that patients might adjust their lifestyle when the disease progresses and have therefore fewer activities that provoke dyspnea than patients with less severe disease. However other studies with appropriate design could answer this important question.

SGRQ and CCQ total scores showed good correlation (rho = 0.769, Table 2) highly indicative of convergent validity. CAT score showed a slightly weaker correlation with SGRQ (rho = 0.646). It is lower than this reported in the study of Jones et al. [10]. The discrepancy of lower correlation between SGRQ and CAT presented in our study could be due to different COPD population studied in terms of severity, gender and nationality. CCQ total score and CAT score also have a strong correlation (rho = 0.644; p < 0.01) supporting the theory that they measure the same construct. However, further studies are needed, including different clinical settings, to confirm the exact magnitude of correlation of CAT with the older quality of life instruments such as the CCQ and the SGRQ.

CAT is a one-dimensional questionnaire and it is very easy in calculation algorithm. In contrast CCQ has more similarities with SGRQ. As the SGRQ the CCQ has a division in domains. In the present study CCQ domains showed a good correlation with the respective SGRQ domains. The advantage of domains is that individual management plans can not only be specified according to the impairment of health status in general but also to the individual domains. A patient with for example an impaired mental state might be managed different from a patient with an impaired functional status. The validity of the CCQ domains is supported by our results that showed that the functional domain of the CCQ correlated significantly with the activity domain of the SGRQ (rho = 0.753; p < 0.01). The Bland and Altman plot (Figure3) shows this high correlation, while the functional status measured by the CCQ is consistently lower than with the SGRQ.

### Longitudinal validity

Overall, health status scores in subjects followed for almost 6 weeks revealed no changes over time. The CAT and CCQ both showed high test-retest reliability, ICC of the CAT was 0.94 and ICC of the CCQ was 0.95 respectively proving that they are both stable over time and supporting their validity to be used in individuals. This study reproduced the results of previous studies, where CAT and CCQ showed a similar high ICC (0.8; CAT) [10] and (0.91-0.99; CCQ) [9,22,23].

The Minimal Clinically Important Difference of the SGRQ is 4 points [24,25], while the MCID for the CAT has not been established officially but was estimated to be around 2 points [26,27]. The MCID of the CCQ has previously been calculated based on three methods and is 0.4 [28]. In our study we were unable to use distribution-based methods to determine and compare the MCID of the three questionnaires. We compared changes in patient reported outcomes scores to measures of variability. The MCID calculated with the SEM of the CCQ and SGRQ is somewhat similar to the MCID’s found in previous studies. The estimated MCID of the CAT, however, was higher 3.76 points. Hence, further studies are needed to determine the MCID of this relatively new tool.

### Strengths and limitations of the study

This is a real life study, the first that did a head to head comparison of CAT, CCQ and SGRQ in three continuous visits. Several other factors were also examined as spirometry, dyspnea, 6MWT and BODE index. This study has some limitations that should be reported. Firstly this study has been limited to one country and performed in one centre. Since no intervention was included many patients showed to be stable over time. This resulted in an unchanged health status making it impossible to calculate the MCID with anchor based methods and to compare the questionnaires responsiveness. Further this study was not designed to see if the CAT and CCQ both reflected indeed all the COPD patient’s relevant aspects. Larger studies with different design could answer this very important issue.

## Conclusion

Our study showed that CAT and CCQ have similar psychometric properties. Compared to the much more often used but rather extensive SGRQ, they are both valid to assess health status. Patients preferred the CCQ since it reflected their status better than the CAT as it had more details on breathing problems which was more important for them than sleep or energy.

## Abbreviations

COPD, Chronic Obstructive Pulmonary Disease; CAT, COPD assessment Test; CCQ, Clinical COPD Questionnnaire; SGRQ, St. George Respiratory Questionnaire; SEM, Standard Error of Measurement; MCID, Minimal Clinical Important Difference; IPCRG, International Primary Care Respiratory Group; BMI, Body mass index; 6MWT, 6-minute walking test; MRC, Medical Research Council dyspnoea scale; BODE, Body mass index, airflow limitation (forced expiratory volume in one second), dyspnoea and 6-min walk distance; GRC, Global Rating of Change scale; ICC, Intraclass Correlation Coefficient.

## Competing interests

Thys van der Molen was involved in the development of both the CCQ (2003) and the CATquestionnaire (2010). All other authors declare no competing interest related to this article.

## Authors’ contributions

IGT, TvdM, and NT participated in the design of the study. IL and JWK performed the statistics.

IGT prepared the first draft. TvdM, NT and NS revised the first draft. All authors have participated in conception and design, helped in drafting, and gave relevant comments. All authors have given their final approval of this version to be published.

## Pre-publication history

The pre-publication history for this paper can be accessed here:

http://www.biomedcentral.com/1471-2466/12/20/prepub
